# Evaluation method of surrounding rock stability: Failure approach index theory of strain limit analysis for engineering applications

**DOI:** 10.1371/journal.pone.0279302

**Published:** 2022-12-22

**Authors:** Lijun Xiong, Haiping Yuan, Hengzhe Li, Yixian Wang, Xiaohu Liu, Chenxu Ye, Wenhui Wang

**Affiliations:** 1 College of Civil Engineering, Hefei University of Technology, Hefei, Anhui, China; 2 College of Civil Engineering and Architecture, Anhui University of Science and Technology, Huainan, Anhui, China; NUST: National University of Sciences and Technology, PAKISTAN

## Abstract

In general, the ultimate parameter selection method of the failure approach index theory among the three-dimensional problems in geotechnical engineering is unclear in theory, and the symbol convention of the failure approach index in engineering calculation is contrary to the stipulation of the numerical simulation software. Hence, the values of the ultimate plastic shear strain are difficult to determine. To solve this problem, the criterion of positive tension and negative compression and the sequence of the principal stress σ_1_ ≤ σ_2_ ≤ σ_3_ are defined in this paper, and the expression of Mohr–Coulomb yield approach index id deduced. Under the condition of the principal strain sequence ε_1_ ≤ ε_2_ ≤ ε_3_, the formula of the ultimate shear strain is derived using the method of the ultimate strain analysis so as to obtain the simple expression and calculation method of the ultimate plastic shear strain, which has provided the calculation parameters for the three-dimensional ultimate plastic shear strain in the Mohr–Coulomb strain softening model and improved the failure approach index theory. In the light of the aforementioned theory, the ultimate strains of cubic concrete specimens are analyzed, and the obtained ultimate strain values are found consistent with previous research findings, which verifies the correctness and reliability of the ultimate strain analysis method. In addition, it is applied to the quantitative elastic–plastic failure analysis of the section coal pillar in Hengjin coal industry for determining its reasonable retainment width. Consequently, the research results can be embraced as the theoretical basis for the stability analysis of geotechnical materials and exhibits engineering application potential.

## 1. Introduction

In recent years, projects like transportation, water and oil transportation pipelines, deep underground disposal of nuclear waste, deep coal mining and, etc. are continuously increasing. Thus, the stability of surrounding rock in underground excavation space must be solved urgently. At this point, the surrounding rock stability evaluation has become a heated issue in academic and engineering circles, and the surrounding rock stability analysis of underground engineering is an important method for comprehensive stability evaluation, as well as an important basis for safety design and construction [1–4]. Furthermore, the failure approach index theory has been adopted by scholars to analyze the stability of surrounding rock. In this way, the concept of the failure approach index proposed by Zhang et al. [5] can effectively evaluate the risk degree of surrounding rock in the elastic stage and the damage degree in the plastic phrase, which is based on the Mohr–Coulomb yield approach index. Its calculation formula can be expressed as:

$$FAI=\left\{\begin{array}{ll} \omega & \left(0\leq \omega <1\right)\\ 1+FD & \left(\omega =1,FD\geq 0\right) \end{array}\right.$$ (1)

Here, FAI refers to the failure approach index; ω represents the complementary parameter of the yield approach index (YAI); and FD signifies the failure degree and is represented by

$$\text{FD}=\gamma^{p}/\gamma_{f}^{p}$$ (2)

Here, *γ*^*p*^ denotes the plastic shear strain, while $\gamma_{f}^{p}$ represents the ultimate plastic shear strain.

When 0 ≤ FAI < 1.0, the surrounding rock is in the elastic stress state; when 1.0 ≤ FAI < 2.0 and FAI ≥ 2.0, it is in the plastic yield and failure state, respectively.

Above all, the failure approach index can accurately and quantitatively evaluate the stability degree of rock mass in each area of surrounding rock and express the location and range of the failure zone, damage zone, and disturbance zone. Moreover, to predict the influence of excavation disturbance on the stability of rock mass in the future, the evolution law of the stability state of rock mass in each zone along with the excavation process can be described as well.

Wang et al. [6] derived the yield approach index function of Hooke-Brown criterion according to the Mohr–Coulomb yield approach index expression. Moreover, the failure degree theory was introduced, and the failure degree concept of coal was defined. Finally, the crack classification method of coal was proposed by dividing the values of the failure approach index. Yao et al. [7] comprehensively and intuitively assessed the stability degree of rock and soil mass in various regions around the shield tunnel running through Hefei Metro Line 1 using the failure approach index theory and quantitatively defined the range of damage area and disturbance area. Zhou et al. [8] evaluated the similarities between the scale model of slope and the prototype failure through the failure approach index theory and proved that the inclined loading method can properly reproduce the occurrence process of landslide. On account of the improved failure approach index, Zhang et al. [9] made a quantitative analysis and assessed the influence degree of the water storage and rainfall in the Three Gorges Reservoir and their combined effects on the bank slope stability of Qianjiangping. Liu et al. [10] employed the failure approach index to quantitatively describe the risk and failure state of surrounding rock and soil mass during the construction of pipe jacking.

Evidently, the application of the failure approach index provides considerable convenience for the stability analysis of geotechnical engineering. However, the formulae for calculating the plastic shear strain and the ultimate plastic shear strain among the failure approach index calculations are complicated and difficult to solve. Meanwhile, the failure approach index represents the stability evaluation theory of surrounding rock based on the Mohr–Coulomb strain softening model, and the simple two-dimensional plastic shear strain *γ*^*p*^ is employed in this calculation formula. With regard to the finite difference software FLAC^3D^, the Mohr–Coulomb strain softening model adopts the three-dimensional plastic shear strain increment Δ*ε*^*ps*^ to calculate the plastic shear strain, which brings crucial difficulties to the three-dimensional numerical simulation. Thereafter, on the basis of prior studies [11, 12], the Mohr–Coulomb strength theory denotes the mathematical expression under the conditions of the criterion of positive compression and negative tension and the sequence of the principal stress *σ*_1_ ≥ *σ*_2_ ≥ *σ*_3_. Nevertheless, with respect to the 3D numerical simulation software, the mechanical symbol convention of positive tension and negative compression and the sequence of the principal stress *σ*_1_ ≤ *σ*_2_ ≤ *σ*_3_ are adopted. For instance, the corresponding function of the Mohr–Coulomb yield approach index and the expression of the ultimate plastic shear strain in the calculation formula of the failure approach index cannot be found in FLAC^3D^ [13] and the discrete element software PFC^3D^ [14].

In this study, the expression of Mohr–Coulomb yield approach index is derived under the conditions of the criterion of positive tension and negative compression and the sequence of the principal stress *σ*_1_ ≤ *σ*_2_ ≤ *σ*_3_. Under the condition of the principal strain sequence *ε*_1_ ≤ *ε*_2_ ≤ *ε*_3_, the analytical expression of the ultimate elastic shear strain and the calculation formula of the ultimate elastic–plastic shear strain are derived so that the simple two-dimensional expression of the ultimate plastic shear strain is obtained. Subsequently, the relationship between the three-dimensional ultimate plastic shear strain and the two-dimensional ultimate plastic shear strain in the Mohr–Coulomb strain softening model are established to determine the critical parameters for the three-dimensional numerical simulation. Eventually, the correctness and reliability of the ultimate strain analysis method are verified by numerical calculation comparison and engineering examples. Therefore, the research findings have developed the theory of the failure approach index, which provides the analytical method and technical reference for the stability analysis of surrounding rock in underground engineering.

## 2. Mohr–Coulomb yield approach index

Sign convention: the positive and negative values of the normal stress refer to the tensile and compressive stresses, respectively (i.e. positive tension and negative compression), and the principal stress order is *σ*_1_ ≤ *σ*_2_ ≤ *σ*_3_. Hence, under the principal stress order of *σ*_1_ ≤ *σ*_2_ ≤ *σ*_3_, the Mohr–Coulomb yield criterion determined by the principal stress can be expressed as:

$$f=\frac{\sigma_{3}-\sigma_{1}}{2}+\frac{\sigma_{3}+\sigma_{1}}{2}\;\text{sin}\;\varphi -c\;\text{cos}\;\varphi =0,$$ (3)

where *σ*_1_ and *σ*_3_ represent the minimum and maximum principal stress, respectively; *c* signifies the cohesion, and *φ* denotes the internal friction angle.

**Fig 1:**
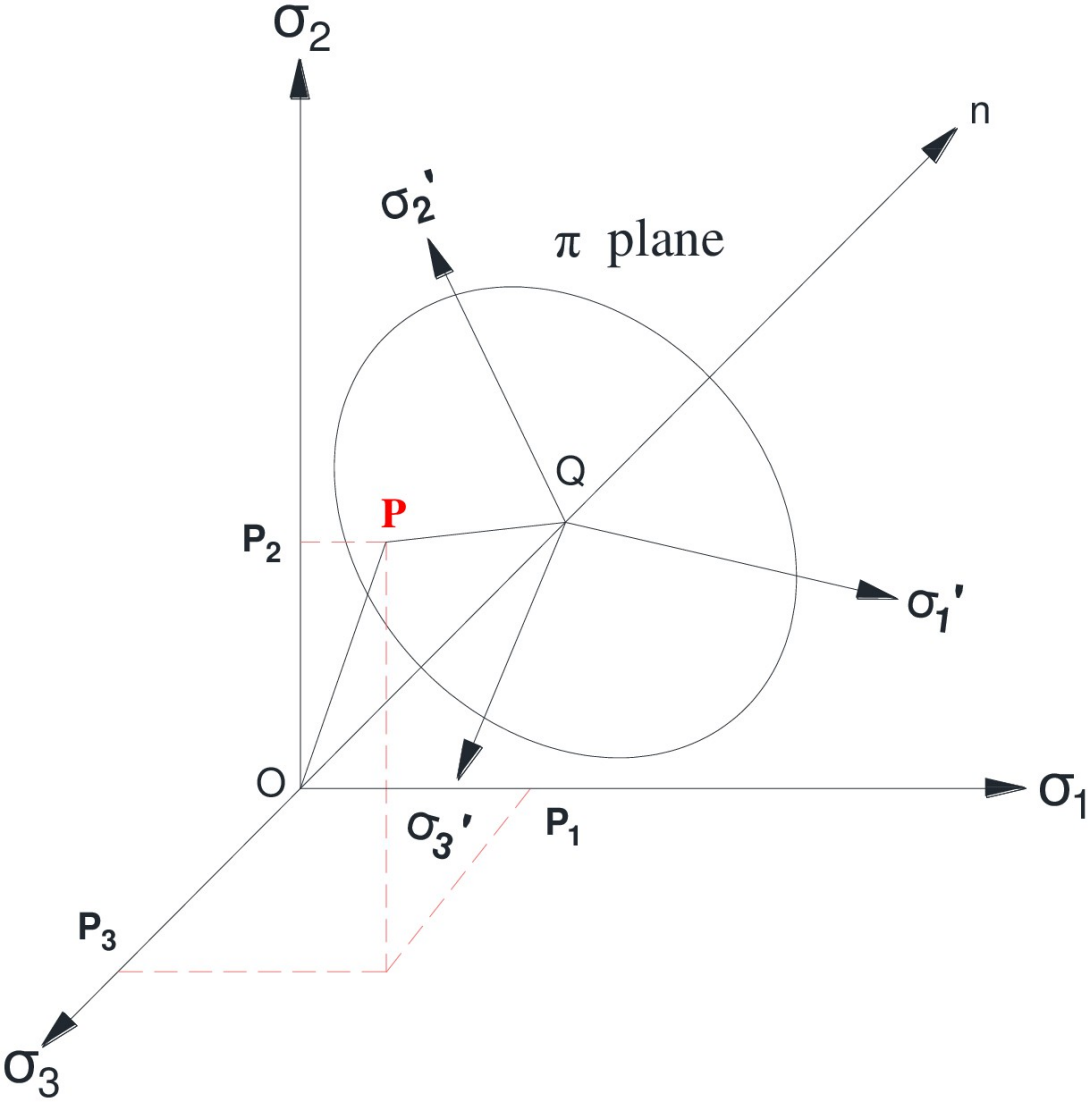
Principal stress space and plane π.

**Fig 2:**
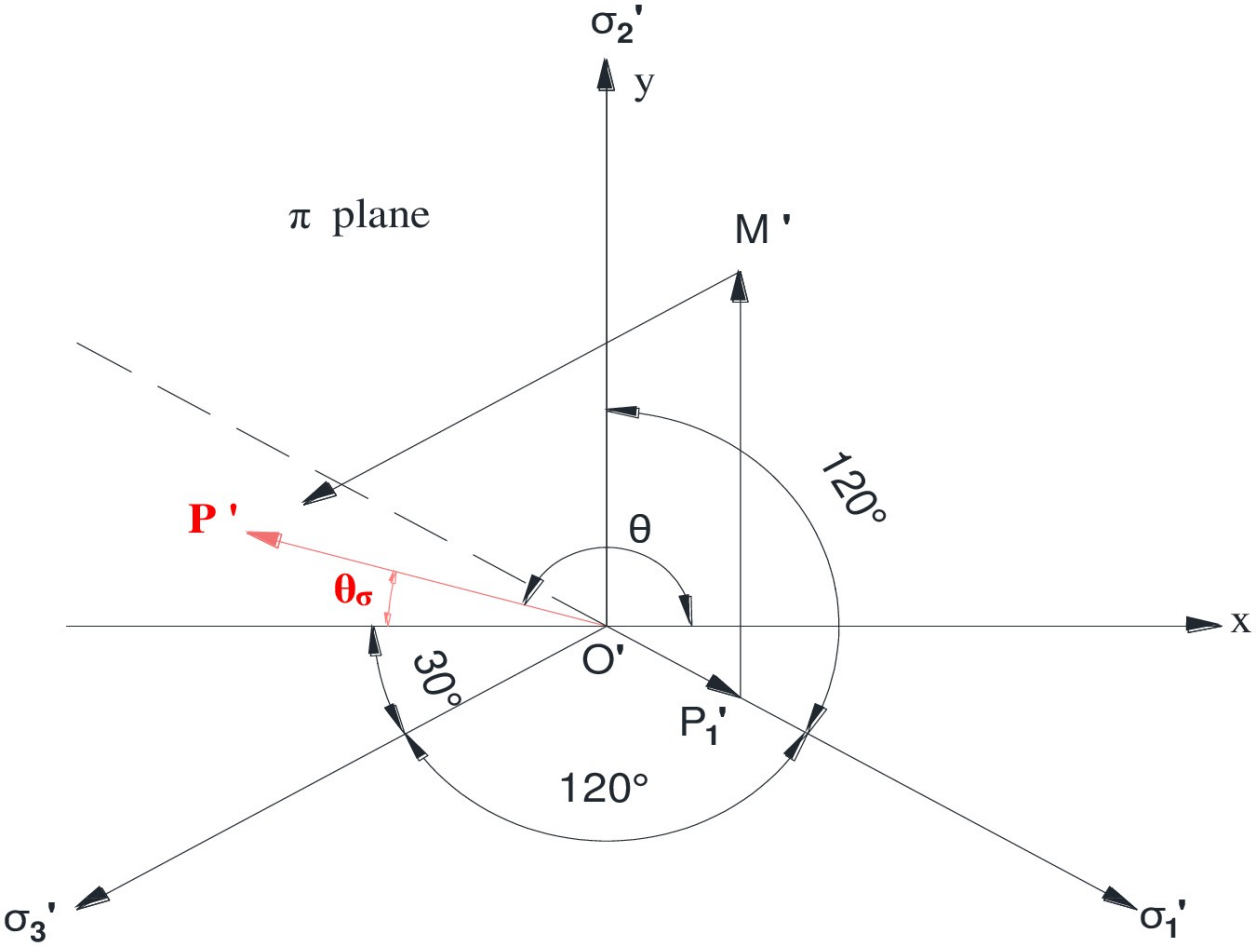
Stress state of a single point on plane π.

Under the conditions of the given principal stress order, the position of the point P in the plane π from the principal stress space is illustrated in Fig 1. According to the results presented in Fig 2, the projections of stress on the plane π on the x-axis and y-axis can be determined as:

$$x=O\prime P_{1}^{\prime }\;\text{cos}30^{{}^{\circ}}-O\prime P_{3}^{\prime }\;\text{cos}30^{{}^{\circ}}=\frac{1}{\sqrt{2}}\left(\sigma_{1}-\sigma_{3}\right)$$ (4)

$$\text{y}=O^{\prime }P_{2}^{\prime }-O^{\prime }P_{1}^{\prime }\;\text{cos}60^{{}^{\circ}}-O^{\prime }P_{3}^{\prime }\;\text{cos}60^{{}^{\circ}}=\frac{1}{\sqrt{6}}\left(2\sigma_{2}-\sigma_{1}-\sigma_{3}\right)$$ (5)

Upon establishing the polar coordinate system on the plane π, the expression of the stress Lord angle is obtained as:

$$\text{tan}\;\theta_{\sigma }=\text{tan}\left(\pi -\theta \right)=-\text{tan}\;\theta =-\frac{y}{x}=\frac{1}{\sqrt{3}}\left(\frac{2\sigma_{2}-\sigma_{1}-\sigma_{3}}{\sigma_{3}-\sigma_{1}}\right),$$ (6)

where *θ*_*σ*_ denotes the stress Lord angle, namely, the included angle between the stress PQ on the plane π and the vertical line of the principal axis $O^{\prime }\sigma_{2}^{\prime }$.

Since the magnitude of the principal stress is irrelevant to the choice of the coordinate system, the expressions of the first invariant of the stress tensor *I*_1_ and the second invariant of the stress deviator *J*_2_ can be represented as:

$$I_{1}=\sigma_{1}+\sigma_{2}+\sigma_{3}$$ (7)

$$J_{2}=\frac{1}{6}\left[{\left(\sigma_{1}-\sigma_{2}\right)}^{2}+{\left(\sigma_{2}-\sigma_{3}\right)}^{2}+{\left(\sigma_{3}-\sigma_{1}\right)}^{2}\right]$$ (8)

Combining Eqs (6), (7) and (8), the expression of the principal stress function, which is signified by the mean principal stress *p*, the generalized shear stress *q* and the stress Lord angle, is obtained as:

$$\left\{\begin{array}{l} \sigma_{1}=\frac{2}{3}q\;\text{sin}\;\left(\theta_{\sigma }-\frac{2}{3}\pi \right)+p\\ \sigma_{2}=\frac{2}{3}q\;\text{sin}\;\theta_{\sigma }+p\\ \sigma_{3}=\frac{2}{3}q\;\text{sin}\;\left(\theta_{\sigma }+\frac{2}{3}\pi \right)+p \end{array}\right.$$ (9)

By substituting the Eq (9) into the Eq (3), which is the yield criterion, denoted by the mean principal stress, the generalized shear stress, and the stress Lord angle, is calculated as:

$$f\left(p,q,\theta_{\sigma }\right)=psin\varphi +\left(cos\theta_{\sigma }/\sqrt{3}-sin\theta_{\sigma }sin\varphi /3\right)q-ccos\varphi =0$$ (10)

**Fig 3:**
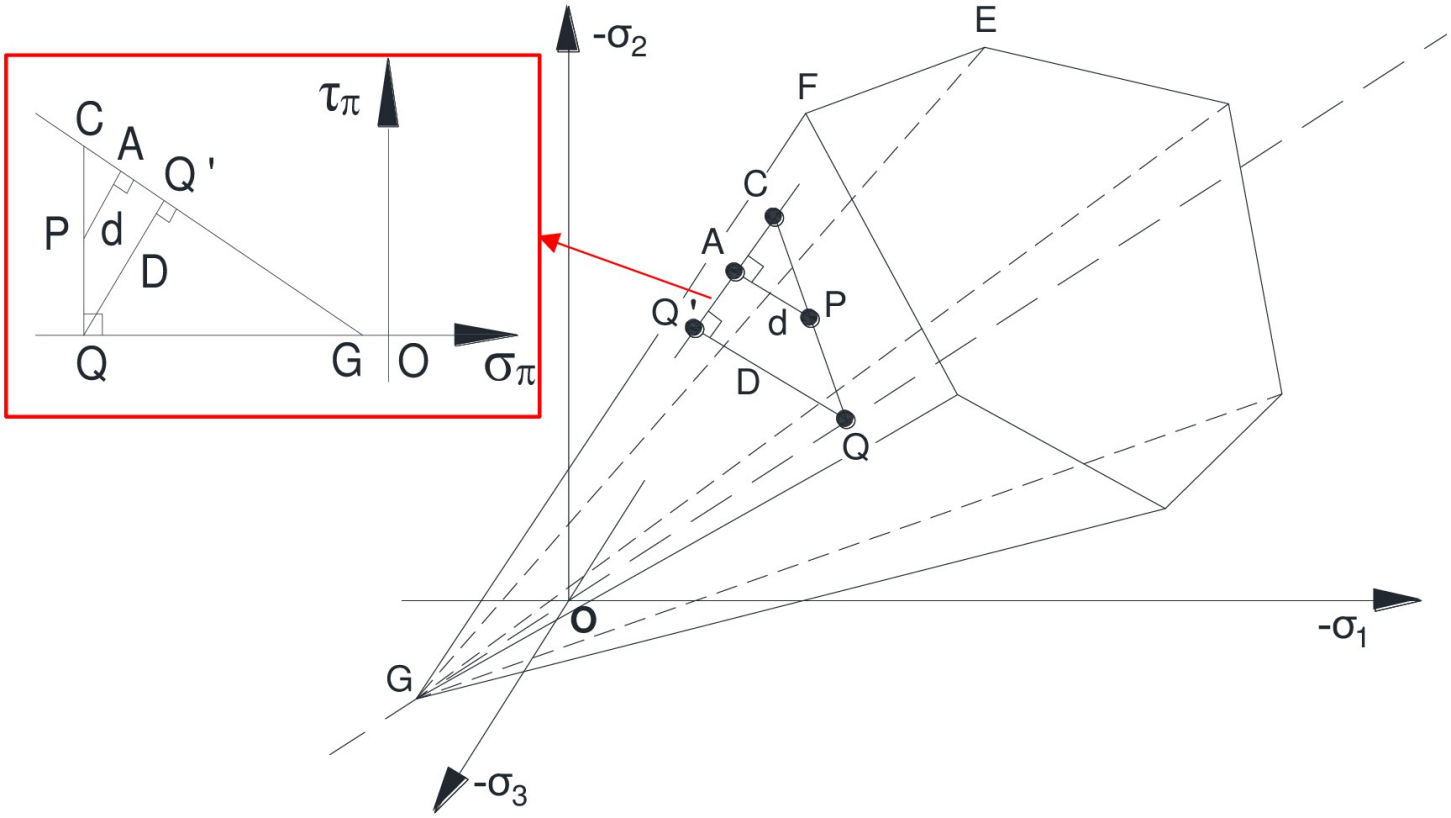
Relationship between the stress point and the yield surface in the principal stress space.

The spatial position relation between the stress point and the yield surface in the principal stress space is depicted in Fig 3. Moreover, the geometric relationship between the stress point P and most stable reference point Q on the meridional plane is illustrated in the left side of Fig 3. Thereinto, the horizontal axis represents the isocline line, and the shear stress on the plane π can be expressed using the vertical coordinate τ_π_ as:

$$\tau_{\pi }=\sqrt{2/3}q$$ (11)

In the principal stress space, the stress state of a point P can be denoted as the point in the meridional plane and plane π. As demonstrated in Fig 3, the line segment *CG* stands for the meridian on the yield plane, and the coordinate values of the points Q, P, and C refer to (*σ*_*π*_, 0), (*σ*_*π*_, *τ*_*π*_), and ($\sigma_{\pi },\;\tau_{\pi }^{\prime }$), respectively.

Since the point C satisfies the Eq (10) concerning the Mohr–Coulomb yield criterion, which is located on the yield surface. Accordingly, the following expression is obtained:

$$\tau_{\pi }^{\prime }=\frac{\sqrt{2}\left(c\;\text{cos}\;\varphi -p\;\text{sin}\;\varphi \right)}{\text{cos}\;\theta_{\sigma }-\text{sin}\;\theta_{\sigma }\;\text{sin}\;\varphi /\sqrt{3}}$$ (12)

With respect to the triangle relationship implied in Fig 3, Δ*PAC* and ΔQQ′*C* are similar to each other. Hence, the yield approach index can be denoted as:

$$YAI=\frac{d}{D}=\frac{L_{PA}}{L_{QQ^{\prime }}}=\frac{L_{PC}}{L_{QC}}=\frac{\tau_{\pi }^{\prime }-\tau_{\pi }}{\tau_{\pi }^{\prime }}=1-\frac{\tau_{\pi }}{\tau_{\pi }^{\prime }}$$ (13)

By substituting the Eqs (11) and (12) into the Eq (13), the expression of the yield approach index under the Mohr–Coulomb yield criterion is attained as:

$$YAI=\frac{p\;\text{sin}\;\varphi +\left(\text{cos}\;\theta_{\sigma }/\sqrt{3}-\text{sin}\;\theta_{\sigma }\;\text{sin}\;\varphi /3\right)q-c\;\text{cos}\;\varphi }{p\;\text{sin}\;\varphi -c\;\text{cos}\;\varphi }$$ (14)

Where: *YAI* refers to the yield approach index.

## 3. Ultimate strain analysis

Rather than considering the deformation and displacement of materials, by solely investigating the material failure of rock and soil masses, the calculation process can be simplified by sidestepping the complex elastic–plastic constitutive models, and the ultimate analysis method of ideal elastic–plastic models can be adopted [15]. In the stress-strain curve of an ideal elastic–plastic material, if it is expressed by stress, the stress is constant and the strain increases infinitely. In this case, the yield and failure criteria are identical, so it is difficult to distinguish the yield and failure state. In terms of the strain expression, the initial yield of the material is defined upon attaining the yield state, which has the ultimate elastic shear strain *γ*_*y*_. At this moment, the material will not be damaged. Nevertheless, with the development of plasticity, the material will enter a state of failure. Thus, the ultimate elastic–plastic shear strain *γ*_*f*_ is attained, i.e., the material encounters the state of the failure strain [16].

### 3.1. Ultimate elastic shear strain

According to the generalized Hooke’s law,

$$\left\{\begin{array}{l} \sigma_{3}-\sigma_{1}=\frac{E}{1+\mu }\left(\varepsilon_{3}-\varepsilon_{1}\right)\\ \sigma_{3}+\sigma_{1}=\frac{E}{1+\mu }\left(\frac{6\mu }{1-2\mu }\varepsilon_{m}+\varepsilon_{3}+\varepsilon_{1}\right), \end{array}\right.$$ (15)

where *E* denotes the elastic modulus, *μ* represents the Poisson’s ratio, and *ε*_*m*_ = (*ε*_1_ + *ε*_2_ + *ε*_3_)/3, which symbolizes the average normal strain.

By substituting the Eq (15) into the Eq (3), the Mohr–Coulomb yield criterion expressed by the principal strain under the principal strain condition of *ε*_1_ ≤ *ε*_2_ ≤ *ε*_3_ can be obtained as:

$$\frac{\varepsilon_{3}-\varepsilon_{1}}{2}+\frac{\varepsilon_{3}+\varepsilon_{1}}{2}\text{sin}\;\varphi =\frac{c\left(1+\mu \right)}{E}\text{cos}\;\varphi -\frac{3\mu }{1-2\mu }\varepsilon_{m}\;\text{sin}\;\varphi$$ (16)

When materials comprising rock and soil masses attain the ultimate elastic state under triaxial stress, their ultimate elastic principal strain should satisfy the following equation:

$$\left\{\begin{array}{l} \varepsilon_{1y}=\frac{1}{E}\left[\sigma_{1}-\mu \left(\sigma_{2}+\sigma_{3}\right)\right]\\ \varepsilon_{3y}=\frac{1}{E}\left[\sigma_{3}-\mu \left(\sigma_{1}+\sigma_{2}\right)\right], \end{array}\right.$$ (17)

where *ε*_1*y*_ and *ε*_3*y*_ denote the minimum and maximum ultimate elastic principal strain, respectively.

By substituting the Eq (17) into the Eq (16), the expression of the ultimate elastic principal strain can be realized as:

$$\left\{\begin{array}{l} \varepsilon_{1y}=-\frac{2c\;\text{cos}\;\varphi }{E\left(1-\text{sin}\;\varphi \right)}+\frac{1+\text{sin}\;\varphi -\mu \left(1-\text{sin}\;\varphi \right)}{E\left(1-\text{sin}\;\varphi \right)}\sigma_{3}-\frac{\mu }{E}\sigma_{2}\\ \varepsilon_{3y}=\frac{2\mu c\;\text{cos}\;\varphi }{E\left(1-\text{sin}\;\varphi \right)}+\frac{1-\text{sin}\;\varphi -\mu \left(1+\text{sin}\;\varphi \right)}{E\left(1-\text{sin}\;\varphi \right)}\sigma_{3}-\frac{\mu }{E}\sigma_{2} \end{array}\right.$$ (18)

On the basis of the condition of the unidirectional stress, the Eq (18) can be simplified as follows:

$$\left\{\begin{array}{l} \varepsilon_{1y}=-\frac{2c\;\text{cos}\;\varphi }{E\left(1-\text{sin}\;\varphi \right)}\\ \varepsilon_{3y}=\frac{2\mu c\;\text{cos}\;\varphi }{E\left(1-\text{sin}\;\varphi \right)} \end{array}\right.$$ (19)

With respect to the derivation of the theoretical model, considering the simplicity of the process and calculation, the ultimate elastic shear strain is expressed as:

$$\gamma_{y}=\frac{2c\left(1+\mu \right)}{E}\sqrt{\frac{1+\text{sin}\;\varphi }{1-\text{sin}\;\varphi }}$$ (20)

### 3.2. Ultimate elastic–plastic shear strain

**Fig 4:**
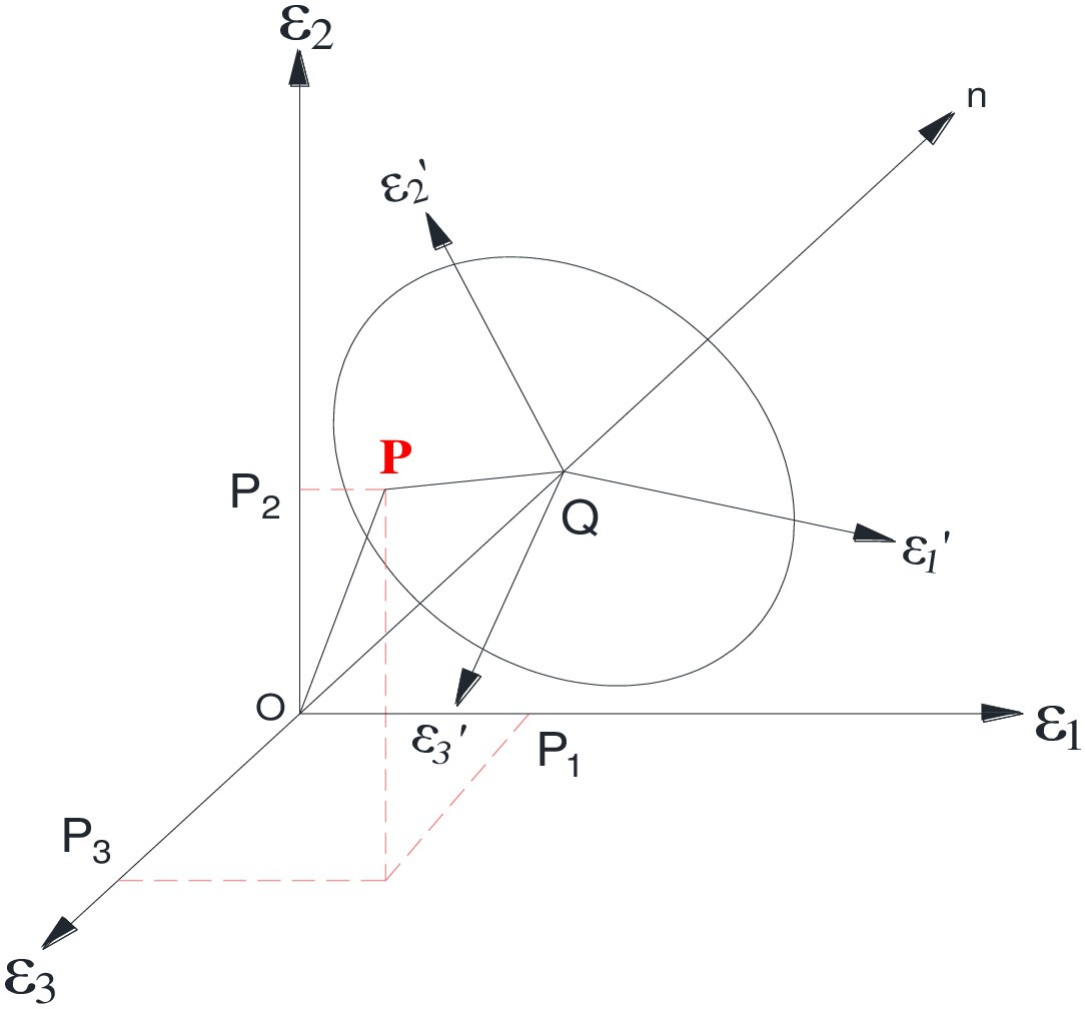
Strain space and strain plane π.

**Fig 5:**
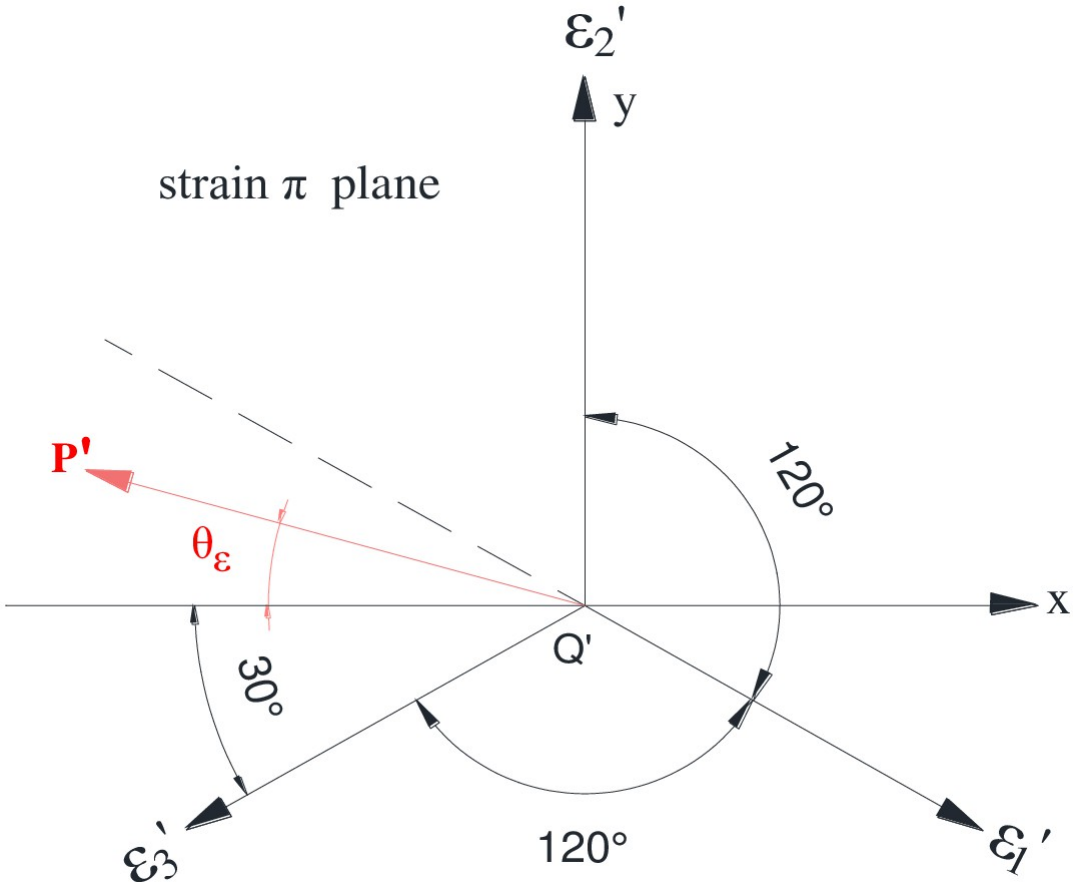
Strain lord angle on plane π.

The strain space, a three-dimensional space comprising three main strains, is used to denote the strain states. As depicted in Fig 4, under the principal strain condition of *ε*_1_ ≤ *ε*_2_ ≤ *ε*_3_, a fixed point in the strain space corresponds to a certain strain state. By virtue of considering the rectangular coordinate O_xy_ on the strain plane π and ensuring that the y-axis direction is consistent with the projection of the axis ε_2_ on the plane π, as illustrated in Fig 5, the following expression is derived:

$$\left\{\begin{array}{l} x=\frac{\varepsilon_{1}-\varepsilon_{3}}{\sqrt{2}}\\ y=\frac{2\varepsilon_{2}-\varepsilon_{1}-\varepsilon_{3}}{\sqrt{6}} \end{array}\right.$$ (21)

Considering the polar coordinate of *r*_*ε*_, *θ*_*ε*_ on the strain plane π, the following representative expression are derived as:

$$r_{\varepsilon }=\sqrt{x^{2}+y^{2}}=\frac{1}{\sqrt{3}}{\left[{\left(\varepsilon_{1}-\varepsilon_{2}\right)}^{2}+{\left(\varepsilon_{2}-\varepsilon_{3}\right)}^{2}+{\left(\varepsilon_{3}-\varepsilon_{1}\right)}^{2}\right]}^{\frac{1}{2}}=\sqrt{2J_{2}^{\prime }},$$ (22)

$$\text{tan}\;\theta_{\varepsilon }=-\frac{y}{x}=\frac{1}{\sqrt{3}}\frac{2\varepsilon_{2}-\varepsilon_{1}-\varepsilon_{3}}{\varepsilon_{3}-\varepsilon_{1}},$$ (23)

where *r*_*ε*_ depicts the strain vector diameter, and *θ*_*ε*_ indicates the strain Lord angle.

Under the condition of principal strain order of *ε*_1_ ≤ *ε*_2_ ≤ *ε*_3_, the principal strain function can be expressed via the variables *r*_*ε*_, *θ*_*ε*_, and ε_m_ as:

$$\left\{\begin{array}{l} \varepsilon_{1}=\sqrt{2/3}r_{\varepsilon }\;\text{sin}\;\left(\theta_{\varepsilon }-\frac{2}{3}\pi \right)+\varepsilon_{m}\\ \varepsilon_{2}=\sqrt{2/3}r_{\varepsilon }\;\text{sin}\;\left(\theta_{\varepsilon }\right)+\varepsilon_{m}\\ \varepsilon_{3}=\sqrt{2/3}r_{\varepsilon }\;\text{sin}\;\left(\theta_{\varepsilon }+\frac{2}{3}\pi \right)+\varepsilon_{m} \end{array}\right.$$ (24)

On the deviator strain plane, the relationship among the principal strain, the shear strain, and the strain Lord angle is expressed as:

$$\varepsilon_{2}-\varepsilon_{m}=\sqrt{\frac{2}{3}}r_{\varepsilon }\;\text{sin}\;\theta_{\varepsilon }=\frac{2}{\sqrt{3}}\sqrt{J_{2}^{\prime }}\;\text{sin}\;\theta_{\varepsilon }$$ (25)

Under the condition of principal strain of *ε*_1_ ≤ *ε*_2_ ≤ *ε*_3_, given the average normal strain *ε*_*m*_ = (*ε*_1_ + *ε*_2_ + *ε*_3_)/3, substituting this expression into Eq (25) yields the expression of the maximum shear strain *γ*_*max*_ as:

$$\gamma_{max}=\varepsilon_{3}-\varepsilon_{1}=2\left(\varepsilon_{3}-\varepsilon_{2}\right)+2\sqrt{3}\sqrt{J_{2}^{\prime }}\;\text{sin}\;\theta_{\varepsilon }$$ (26)

In terms of the unidirectional stress, the isotropic material, and the constant Poisson’s ratio, the lateral strain of the material will be identical. In this case, the strain Lord angle *θ*_*ε*_ = 30°, which is substituted into the Eq (26), and the relationship between the ultimate elastic–plastic shear strain of the material and the ultimate elastic–plastic principal strain can be obtained as:

$$\gamma_{f}=\sqrt{3J_{2f}^{\prime }}=\varepsilon_{3f}-\varepsilon_{1f},$$ (27)

where *γ*_*f*_ represents the ultimate elastic–plastic shear strain, and *ε*_3*f*_ and *ε*_1*f*_ symbolize the ultimate strain values of the elastic–plastic principal strain of *ε*_3_ and *ε*_1_.

The numerical solution of the ultimate elastic–plastic shear strain can be obtained via the numerical method of the ultimate strain analysis. Upon implementing the numerical ultimate analysis method, a point failure will occur when a certain element in the material model attains the state of the ultimate plastic shear strain limit. Furthermore, if the shear strain of the material model at all points on the penetrating failure plane is greater than the ultimate plastic shear strain, the overall failure state of the material model will emerge, and the penetration of the ultimate strain zone represents the sufficient and necessary condition for the overall failure state of the material model [17].

### 3.3. Ultimate plastic shear strain

The failure and yield surface of the Mohr–Coulomb possess the same shape and size, an unequal-angle hexagonal cone, with an unequal-angle hexagon on the partial plane, and the distance between the center of failure surface and center of yield surface is equal to $\gamma_{f}^{p}$ [18]. According to the ideal elastic–plastic model, the expression of the ultimate plastic shear strain can be represented as:

$$\gamma_{f}^{p}=\gamma_{f}-\gamma_{y}$$ (28)

In relation to the Mohr–Coulomb’s strain-softening model, the plastic parameters are introduced to account for the dependence of the loss of rock strength on plastic deformation. Generally, common plastic parameters are in two forms: the form of the internal variable, namely, *γ*^*p*^, and the the form of the plastic strain increment, i.e. Δ*ε*^*ps*^.

Under the condition of principal strain order of *ε*_1_ ≤ *ε*_2_ ≤ *ε*_3_, the two-dimensional plastic shear strain can be expressed as [19]:

$$\gamma^{p}=\varepsilon_{3}^{ps}-\varepsilon_{1}^{ps},$$ (29)

where *γ*^*p*^ represents the two-dimensional plastic shear strain, and $\varepsilon_{3}^{ps}$ and $\varepsilon_{1}^{ps}$ signify the maximum and the minimum plastic principal strain, respectively.

In the Mohr–Coulomb’s strain-softening model, the three-dimensional plastic shear strain is adopted [19, 20], whose incremental form is presented as follows:

$$\Delta \varepsilon^{ps}=\frac{\sqrt{{\left(\Delta \varepsilon_{3}^{ps}-\Delta \varepsilon_{m}^{ps}\right)}^{2}+{\left(\Delta \varepsilon_{m}^{ps}\right)}^{2}+{\left(\Delta \varepsilon_{1}^{ps}-\Delta \varepsilon_{m}^{ps}\right)}^{2}}}{\sqrt{2}},$$ (30)

where

$$\Delta \varepsilon_{m}^{ps}=\left(\Delta \varepsilon_{1}^{ps}+\Delta \varepsilon_{3}^{ps}\right)/3.$$ (31)

As for the finite difference software, FLAC^3D^, the strain-softening model based on the Mohr–Coulomb criterion adopts the three-dimensional plastic shear strain increment. Moreover, establishing a relationship between the internal variable, *γ*^*p*^, and the three-dimensional plastic shear strain, Δ*ε*^*ps*^, in the strain-softening model is essential for embedding the failue approach index formulation into the FLAC^3D^ software for numerical calculation. The Mohr–Coulomb plastic potential function and yield function have similar expressions. Under the condition of principal stress of *σ*_1_ ≤ *σ*_2_ ≤ *σ*_3_, the shear plastic potential function can be expressed as

$$g^{s}=\sigma_{3}-\kappa \sigma_{1},$$ (32)

where

$$\kappa =\left(1-\text{sin}\;\psi \right)/\left(1+\text{sin}\;\psi \right),$$ (33)

where *g*^*s*^ represents the shear plastic potential function, and *ψ* denotes the dilatancy angle.

In the Mohr–Coulomb’s strain-softening model, the shear plastic potential function corresponds to the law of uncorrelated flow, which is expressed as:

$$\Delta \varepsilon_{i}^{ps}=\lambda^{s}\frac{\partial g^{s}}{\partial \sigma_{i}}\;\left(i=1,3\right),$$ (34)

where *λ*^s^ denotes the plastic factor.

According to the Eq (34), the relationship between the principal strain increments $\Delta \varepsilon_{3}^{ps}$ and $\Delta \varepsilon_{1}^{ps}$ is established, which can be signified as:

$$\Delta \varepsilon_{1}^{ps}=-\kappa \Delta \varepsilon_{3}^{ps}$$ (35)

In accordance with the Eqs (31) and (35), the Eq (30) can be simplified to the following:

$$\Delta \varepsilon^{ps}=\frac{\sqrt{3}}{3}\sqrt{1+\kappa +\kappa^{2}}\Delta \varepsilon_{3}^{ps}$$ (36)

In case of a constant dilatancy angle, the relationship between the three-dimensional plastic shear strain, *ε*^*ps*^, and the two-dimensional plastic shear strain, *γ*^*p*^, in Mohr–Coulomb’s strain-softening model can be denoted as:

$$\sum \Delta \varepsilon^{ps}=\frac{\sqrt{3}}{3}\sqrt{1+\kappa +\kappa^{2}}\sum \Delta \varepsilon_{3}^{ps}$$ (37)

$$\varepsilon^{ps}=\frac{\sqrt{3}}{3}\sqrt{1+\kappa +\kappa^{2}}\varepsilon_{3}^{ps}$$ (38)

Based on the Eq (35), the Eq (38) can be rewritten as:

$$\varepsilon^{ps}=-\frac{\sqrt{3}}{3\kappa }\sqrt{1+\kappa +\kappa^{2}}\varepsilon_{1}^{ps}$$ (39)

With regard to the relationship between the Eqs (38) and (39), upon combining with the Eq (29), the three-dimensional plastic shear strain can be represented as:

$$\varepsilon^{ps}=\frac{\sqrt{3}}{3}\sqrt{1+\kappa +\kappa^{2}}\frac{\gamma^{p}}{1+\kappa }$$ (40)

Assuming that the dilatancy angle *ψ* = 0, the Eq (40) can be simplified as:

$$\varepsilon^{ps}=\gamma^{p}/2$$ (41)

In line with the ultimate strain state, the three-dimensional ultimate plastic shear strain in the Mohr–Coulomb’ strain-softening model is expressed as:

$$\varepsilon_{f}^{ps}=\frac{1}{2}\gamma_{f}^{p}=\frac{1}{2}\left(\gamma_{f}-\gamma_{y}\right)$$ (42)

### 3.4. Solution and verification of ultimate strain

**Fig 6:**
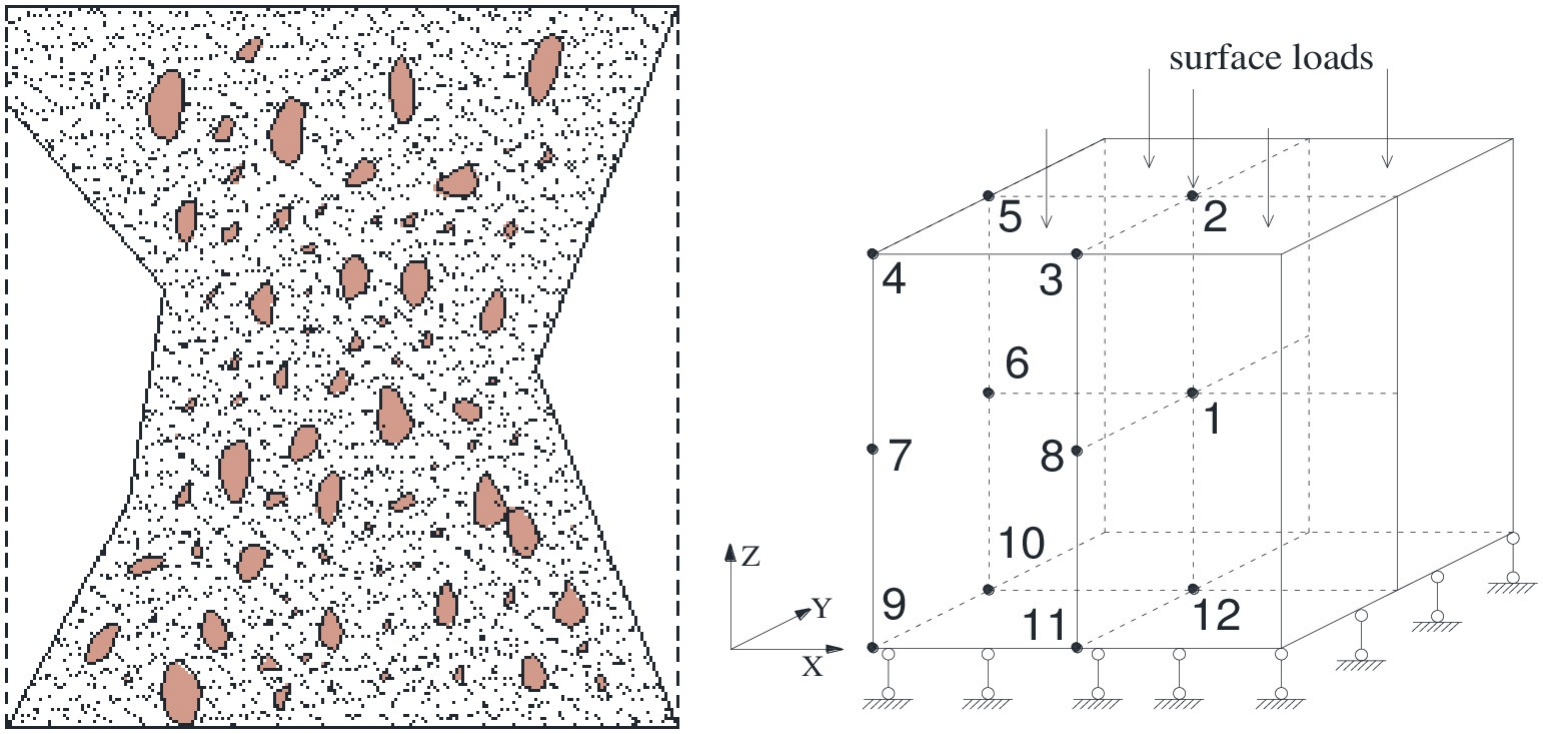
Failure mode and calculation model of the concrete specimen.

In this study, the numerical ultimate analysis of C20 cube concrete specimen is carried out by FLAC^3D^. The geometric size of the calculation model is 150 mm×150 mm×150 mm. Each side is divided into 19 grids, with a total of 6859 elements and 8000 nodes. Three direction constraints are applied to the bottom surface of the model, and uniform vertical load downward is applied to the top surface without considering the friction of the top surface. According to the failure form of the cube concrete specimen, there are 12 key recording points (units) in the calculation model, as shown in Fig 6. In addition, the physical and mechanical parameters of C20 concrete are listed in Table 1.

**Table 1:** Physical and mechanical parameters of C20-grade concrete [21].

Concrete	Density *ρ*/(kg/m^3^)	Elastic Modulus *E*/(GPa)	Possion’s ratio *μ*	Cohesion *c*/(MPa)	Angle of internal friction *φ*/(°)	Tensile strength *σ*_*t*_/(MPa)
C20	2400	25.5	0.2	2.6	61.6	1.54

Through the numerical simulation analysis, several criterion methods for failure have been proposed [22, 23], and the method of iterative non-convergence for failure estimation has been employed in this study. In the calculation process, the axial load of the model is continuously increased until the model approaches the state of ultimate failure, at which point the load signifies the ultimate load.

**Fig 7:**
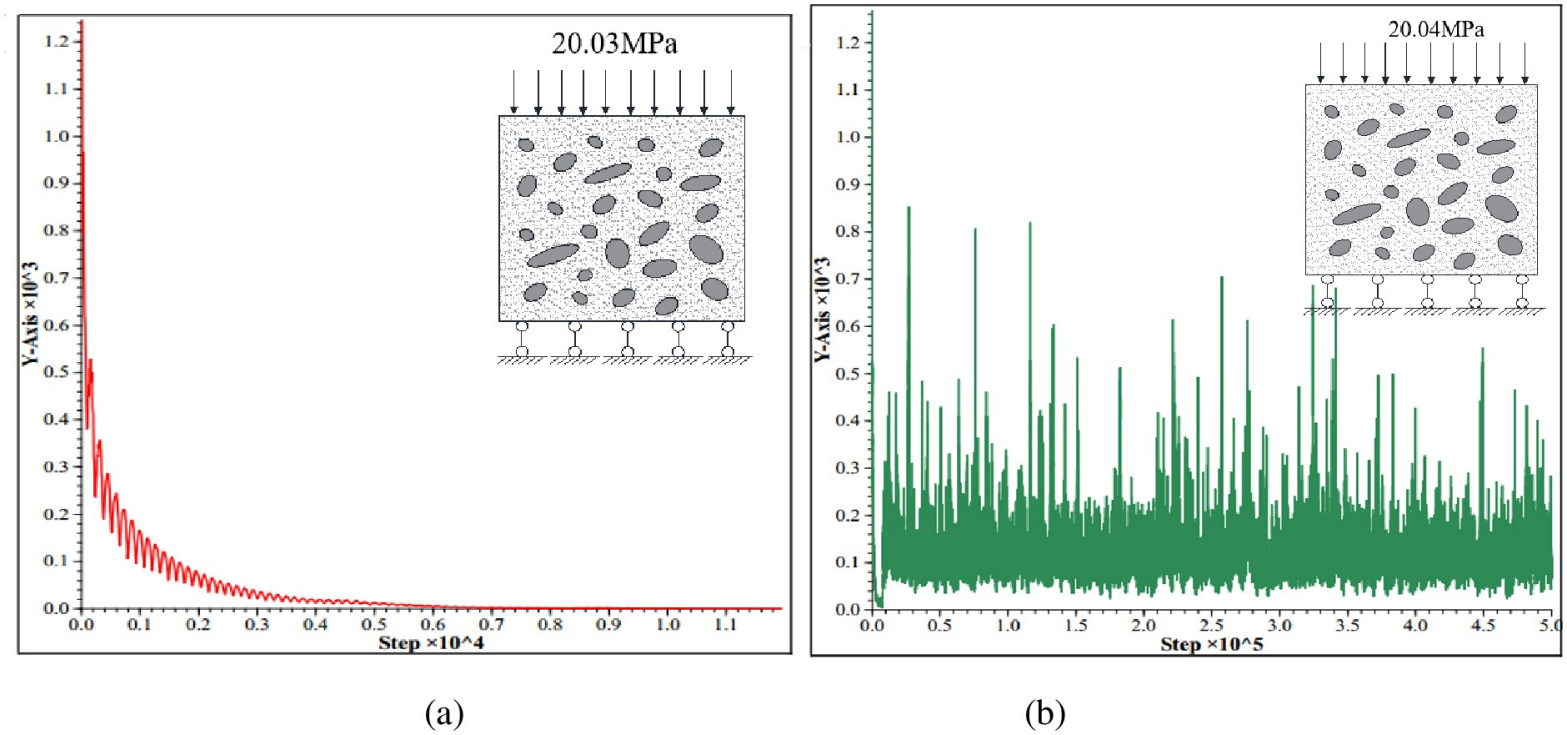
Maximum unbalanced force curve.

Fig 7(a) depicts the maximum unbalanced force curve for a load of 20.03 MPa. Evidently, the latter section of the convergence curve presents a horizontal straight line and tends to be zero, indicating that the calculation converges and the model will not be damaged. Similarly, the maximum unbalanced force curve for a load of 20.04 MPa is depicted in Fig 7(b): the latter part of the curve fluctuates continuosuly. In particular, observing the convergance trend during the calculation is a complicated task, implying that the model will be damaged.

Therefore, the ultimate load onto the concrete cube specimen is 20.03 MPa, which is consistent with the strength grade of the C20-grade concrete, and this result indicates that the given shear strength parameters of concrete are accurate and reliable.

Generally, the elastic strain of concrete is linearly related to the stress level and can be obtained directly using Hooke’s law. Under the condition of principal strain sequence of *ε*_1_ ≤ *ε*_2_ ≤ *ε*_3_, the ultimate elastic principal strain and ultimate elastic shear strain of concrete can be calculated using the Eqs (19) and (20), respectively, and the values of the calculated ultimate strain are enumerated in Table 2.

**Table 2:** Ultimate strain values of the axial, lateral and shear strains of C20-grade concrete.

Concrete	Compressive strength *σ*_*c*_/ (MPa)	Ultimate elastic principal strain /‰		Ultimate elastic–plastic principal strain /‰		Ultimate shear strain/‰		
		Axial *ε*_1*y*_	Lateral *ε*_3*y*_	Axial *ε*_1*y*_	Lateral *ε*_3*y*_	Elastic *γ*_*y*_	Elastic–plastic *γ*_*f*_	Plastic $\gamma_{f}^{p}$
C20	20.03	−0.8	0.159	−1.36	0.512	0.96	1.87	0.91

**Fig 8:**
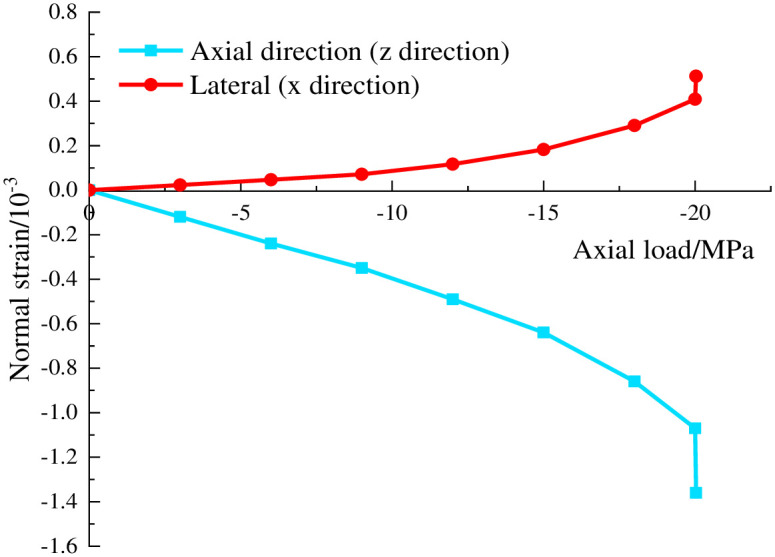
Axial load–principal strain curve of C20-grade concrete.

Concrete is regarded as an ideal elastoplastic material, and the ultimate strain values of 12 elements in the model are calculated under the ultimate load. During the numerical calculation, the development of the strain of unit 5, which is onto the middle edge of the top surface of the model, was evident. When the model is cycled to the default maximum unbalanced force ratio, the strain of the unit 5 approaches the maximum level and failure will inevitably occur. The curve of the axial load and principal strain are exhibited in Fig 8. As the unit is located at the central portion of the top surface of the cube, the failure of the unit will inevitably lead to overall failure of the cement-based concrete cube specimen. Notably, this element strain represents the ultimate strain of the C20-grade concrete. Under the condition of principal strain order of *ε*_1_ ≤ *ε*_2_ ≤ *ε*_3_, the ultimate elastoplastic strains of concrete are listed in Table 2.

The ultimate plastic shear strain of concrete is calculated according to the Eq (28), whose value is displayed in Table 2.

The ultimate strain values of concrete, as summarized in the monograph “Concrete Structure Design Principle”, are listed in Table 3 [24]. On the basis of the condition of principal strain order of *ε*_1_ ≤ *ε*_2_ ≤ *ε*_3_, the ultimate strain values of concrete calculated by the aforementioned formulas are consistent with those in Table 3, which verifies the correctness of the ultimate strain analysis and reliability of the numerical ultimate analysis method.

**Table 3:** Ultimate elastic–plastic strain of C20-grade concrete.

Concrete	Compressive strength *σ*_*c*_/ (MPa)	Ultimate elastic principal strain *ε*_1*y*_/‰	Ultimate Elastic–plastic principal strain *ε*_1*f*_/‰	Ultimate elastic shear strain *γ*_*y*_/‰	Ultimate elastic–plastic shear strain *γ*_*f*_/‰	Ultimate Plastic shear strain $\gamma_{f}^{p}$/‰
C20	20.03	−0.79	−1.37	0.95	1.85	0.90

## 4. Application of failure approach index theory of ultimate strain analysis to stability analysis of coal pillar

The 9–10 coal seam of Linfen Tianyu Hengjin Coal Industry Co., Ltd., Anhui North Coal and Electricity Group, is commercially mined; the average thickness of the mine is 5.6 m, and the dip angle is 3–6º, which corresponds to that of a near-horizontal type. Moreover, the ground elevation ranges from +1397 m to +1424 m, and the mining upper- and lower-limit elevation is +1043.32 m and +983.34m, respectively. During the extraction process, the width of the coal pillar in the remaining section is approximately 30 m as yet, which is the traditional empirical width. Considering the geological conditions of the shallow buried depth, thick coal seam, and hard roof, this wide coal pillar will cause the waste of resources, and thus, the stability of narrow coal pillar should be investigated from a roadway protection perspective to reduce the wastage of coal resources. Hence, the determination of a reasonable width for coal pillars is of great significance to the coal mining industry in this area.

In this study, the lateral driving roadway of 9101 goaf in Xiyi mining area is considered as the research object to investigate the stability of the section coal pillar. The length of the 9101 working face is 120 m, and the main roof is constituted of K2 limestone with an average thickness of 13.09 m. Additionally, the immediate floor is mudstone, and the local face comprises sandy mudstone with an average thickness of 1.82 m. Moreover, the main floor is mainly comprises aluminum mudstone and limestone with an average thickness of 12.71 m. The tunnel section (length: 4 m; height: 2.5 m) represents a rectangular form. The physical and mechanical parameters of each rock layer are summarized in Table 4.

**Table 4:** Physical and mechanical parameters of each rock layer [25].

Lithology	Density (kg/m^3^)	Elastic Modulus (GPa)	Possion’s ratio	Cohesion (MPa)	Internal friction angle (°)	Tesnile strength (MPa)
main roof	2580	40.16	0.21	5.46	40	9.8
9–10 coal seam	1400	3.80	0.21	1.46	28	0.8
immediate floor	2230	4.65	0.2	1.85	30	1.1
main floor	2873	38.7	0.28	4.2	42	3.3

**Fig 9:**
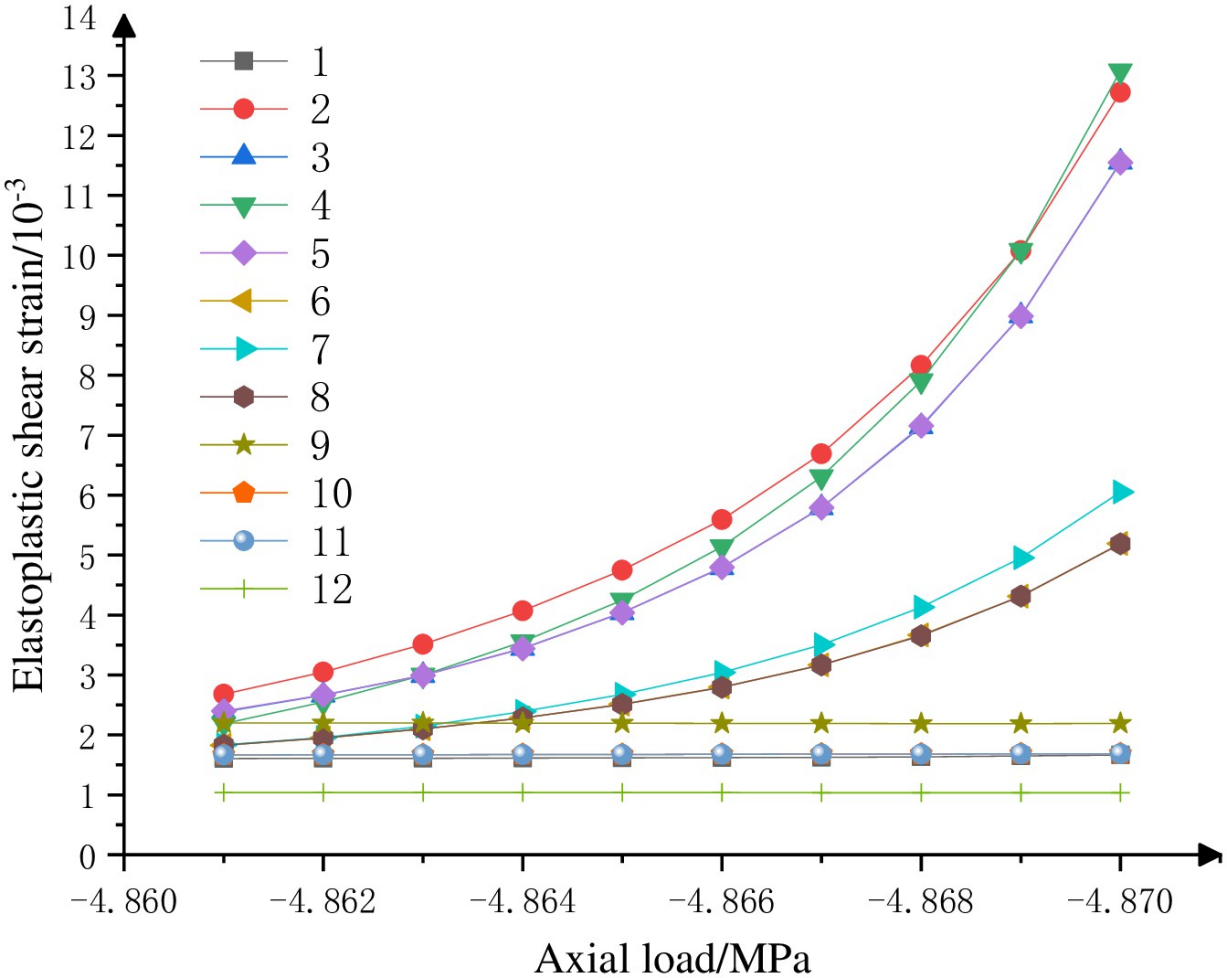
Relationship between near-ultimate axial load and elastoplastic shear strain.

By means of the Eq (20), the ultimate elastic shear strain is calculated to be 0.16%. Obtained via the numerical analysis of ultimate strain, the 12 key units of the elastic and plastic shear strain curve in the calculation model are exhibited in Fig 9. Notably, the elastic and plastic shear strain of the unit 4 is developing promptly, which is located in the upper specimen. In this case, the failure of the unit is bound to result in the overall failure of the specimen. Thus, the strain of this unit denotes the ultimate elastoplastic shear strain, whose value is equal to 1.3%. Accordingly, the ultimate plastic shear strain of the coal pillar is 1.15%. According to the Eq (42), the three-dimensional plastic shear strain value of the Mohr–Coulomb’s strain-softening model is 0.0057, which is adopted in the FLAC^3D^ software for the numerical simulation. Ultimately, the elastoplastic quantitative value of the section coal pillar can be obtained using the failure approach index theory, thereby yielding an updated method for determining the reasonable width of the section coal pillar.

**Fig 10:**
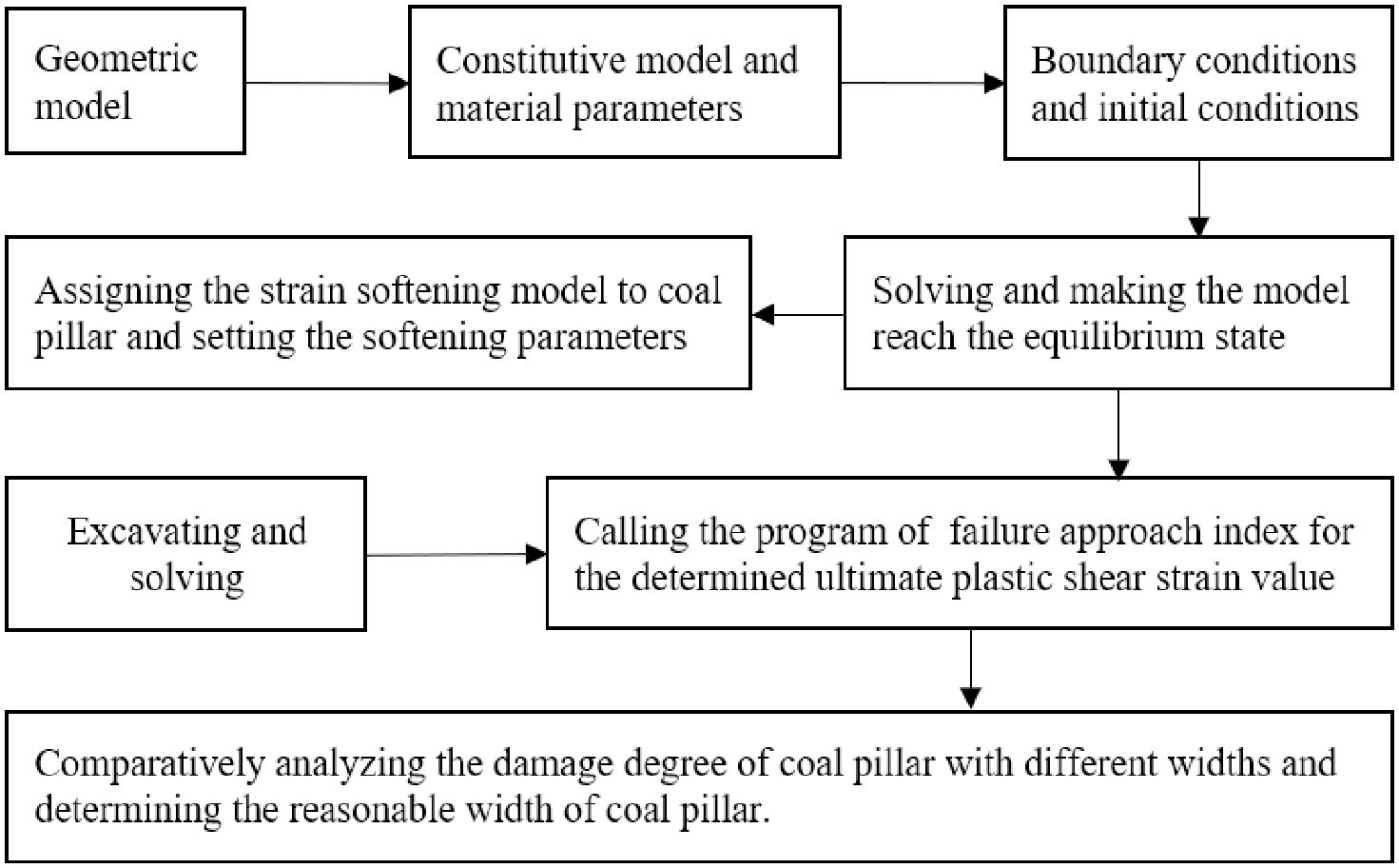
Numerical simulation flow chart.

On account of the aforementioned ultimate strain analysis, the ultimate plastic shear strain value of the coal pillar is determined, which provides critical values for the calculation of failure approach index. The numerical simulation flow chart of the failure approach index theory of the ultimate strain analysis in section coal pillar stability analysis is displayed in Fig 10. Evidently, there are 119611, 118586, and 125592 nodes, and 693631, 688130, and 729879 units of the coal pillar model for widths of 5, 7, and 9 m, respectively. In addition, the boundary conditions of the coal pillar model signify that the vertical downward uniform load is imposed on the top surface, and the other five surfaces are fixed.

**Fig 11:**
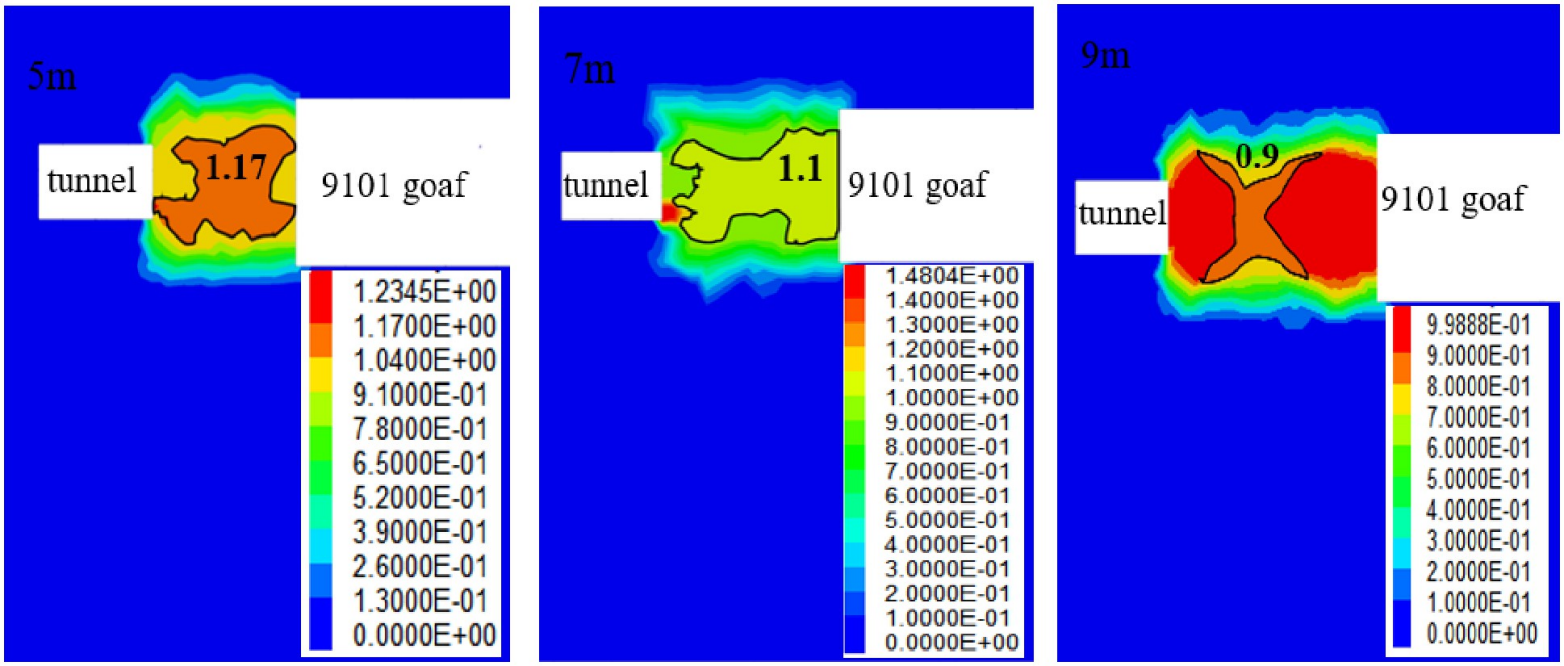
Cloud diagram of the failure approach index of the section coal pillars.

In the coal pillar bearing core area, the failure approach index of the three schemes is 1.17, 1.1, and 0.9, as demonstrated in Fig 11. According to the elastic kernel theory of the coal pillars [26] and the failure approach index partition [5], the width of the coal pillar for the failure approach index of 0.9 is considered the reasonable width of the coal pillar in the reserved section. Therefore, the width of the coal pillars in Xiyi mining area is approximately 9 m.

**Fig 12:**
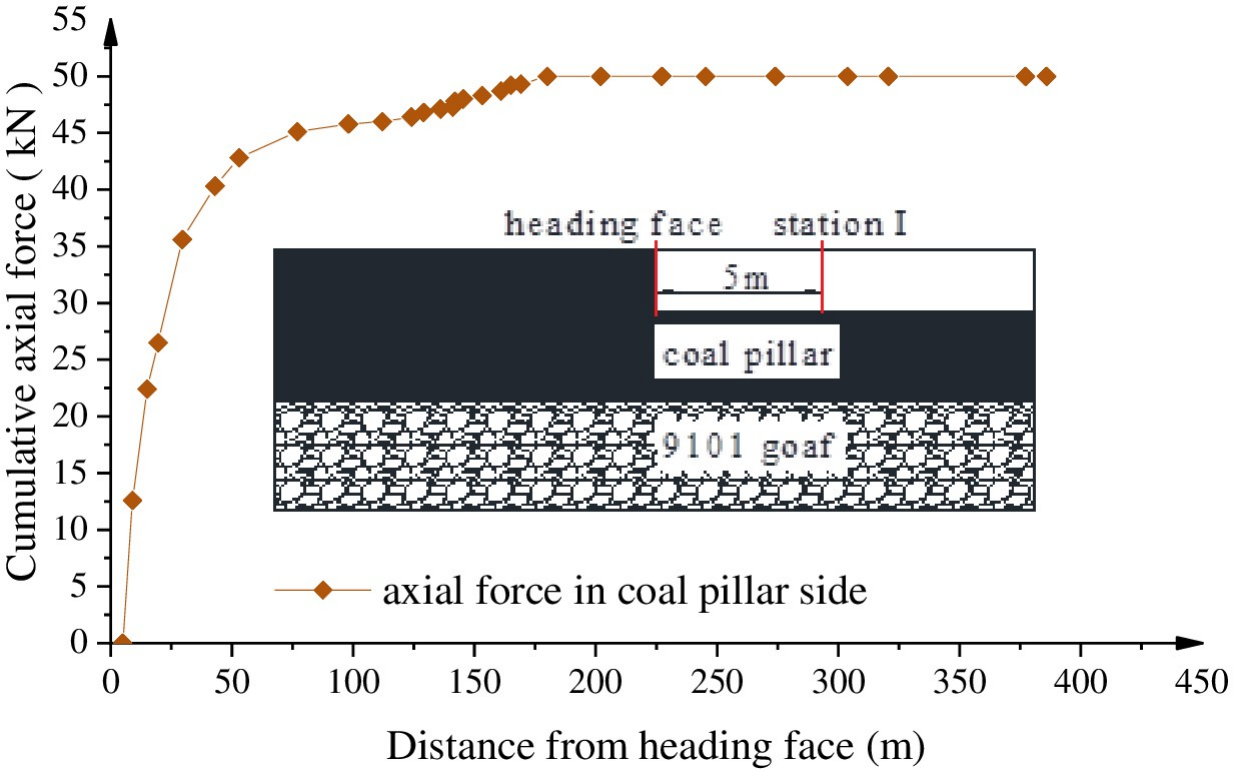
Monitoring section layout diagram and axial force variation diagram of achor of the observation station I.

**Fig 13:**
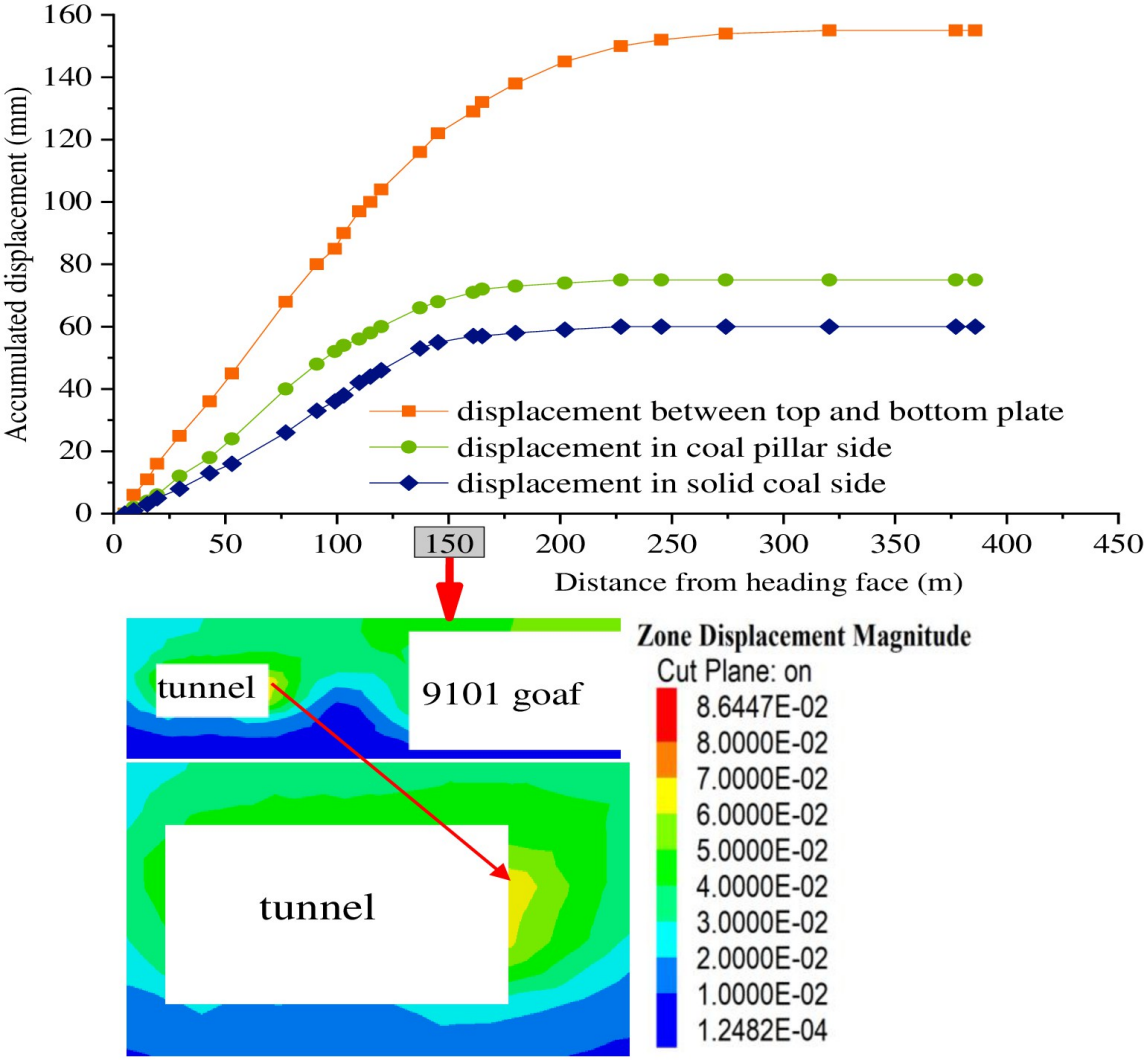
Cloud image of the amount of the displacement deformation and numerical displacement of coal pillar side of roadway surface of the observation station I.

To verify the own stability of width with 9 m of coal pillar in Xiyi mining area, a monitoring station on Section I of surface displacement and anchor axial force is arranged at a distance of 5 m from the roadway head during the excavation of the mining roadway. By virtue of monitoring the force change of coal pillar and observing the deformation of the roof and floor of the roadway and the two sides of the roadway, the stability of the coal pillar is fed back, and the rationality of the width of the coal pillar is judged. As indicated by Fig 12, the axial force of the bolt increases rapidly when the roadway is pushed about 20 m, and the change rate of the axial force of the bolt slows down significantly when the roadway is pushed about 100 m from the head. At 150 m from the head, the axial force of the bolt gradually becomes stable. As can be observed from Fig 13, the cumulative displacement of the top and bottom plate of the measuring station I is 155 mm, that of the solid coal side is 60 mm, and that of the coal pillar side is 75 mm, which is consistent with the numerical displacement nebulogram of the coal pillar side. With the advance of roadway driving, the influence of roadway driving on the measuring station I gradually decreases, whose deformation and deformation rate tend to be stable, the roadway rock pressure is not proved to be apparent, and the surrounding rock stability of the roadway performs preferable. Consequently, the research results of axial force and surface displacement of anchor bolt in station I indicate that it is reasonable to reserve the section with coal pillar of width of 9 m.

## 5. Discussion

Currently, the rapid development of the ultimate analysis and numerical ultimate analysis methods have provided a convenient way to solve the ultimate strain of geotechnical materials, whose reliability and adaptability have also been demonstrated [27, 28]. The failure approach index theory of the ultimate strain analysis proposed in this paper offers a simple expression of the ultimate plastic shear strain and solves the problem of the value of the ultimate plastic shear strain through the three-dimensional numerical simulation of the failure approach index, which provides convenient conditions for the broader application of the failure approach index and improves and develops the theory of the failure approach index.

Herein, the ultimate shear strain refers to the expression obtained under the condition of unidirectional force. If considering the triaxial stress or anisotropy of rock and soil materials, the methodology in this paper can be improved. However, the expression will evidently be more complicated, and the solution will become more difficult. Meanwhile, the failure approach index theory of the ultimate strain analysis put forward in this study can be applied to rock underground engineering, whose application scope needs to be further expanded. It can also be applied to pipeline engineering, subway tunnel engineering, deep underground disposal storage of nuclear waste and other construction projects for extending the application scope and examining its applicability.

## 6. Conclusions

1. Notably, the Mohr–Coulomb yield criterion in the principal stress space is derived under the conditions of positive tension and negative compression and the sequence of the principal stress *σ*_1_ ≤ *σ*_2_ ≤ *σ*_3_. Moreover, the expression of the Mohr–Coulomb yield approach index is obtained according to the spatial geometric relation.
2. Under the principal strain sequence *ε*_1_ ≤ *ε*_2_ ≤ *ε*_3_ and the condition of the unidirectional force, the analytical expression of the ultimate elastic shear strain is derived, and the relationship between the ultimate elastic–plastic shear strain and the principal strain is acquired. Then, the two-dimensional expression of the ultimate plastic shear strain is obtained. Furthermore, the relationship between the three-dimensional plastic shear strain and the two-dimensional plastic shear strain in the Mohr–Coulomb strain softening model is established so that the calculation parameters of the three-dimensional ultimate plastic shear strain in the Mohr–Coulomb strain softening model can be determined and the failure approach index theory can be improved.
3. The ultimate strain analysis of specimen of concrete C20 is carried out through the derived theoretical formulae, and the obtained ultimate strain values are found to be consistent with the previous research findings, which verifies the correctness and reliability of the ultimate strain analysis method.
4. The failure approach index theory of the ultimate strain analysis is applied to the quantitative elastic–plastic analysis of the section coal pillar in the section of Hengjin coal industry. The reasonable retainment width of the section coal pillar is calculated as 9 m, which is shown to be rational by using the feedback of the failure approach index theory from the monitoring results of roadway mine pressure.

## Supporting information

S1 Table Table data of axial load and principal strain of C20-grade concrete and the one of axial load and elastic-plastic shear strain of 12 key elements of coal pillar specimen. (DOCX)
