# Supplementary material for: Evaluation method of surrounding rock stability: Failure approach index theory of strain limit analysis for engineering applications
Source: PLoS One. 2022 Dec 22;17(12):e0279302. doi: 10.1371/journal.pone.0279302 (PMC9778588; doi:10.1371/journal.pone.0279302)
Supplement: S1 Table — (DOCX) [file pone.0279302.s001.docx]

Table Data of axial load and principal strain of C20-grade concrete and the one of axial load and elastic-plastic shear strain of 12 key elements of coal pillar specimen

| **Axial Load/**MPa | **𝜀_1f_** /‰ | **𝜀_3f_** /‰ |
| --- | --- | --- |
| 0.00 | 0.000 | 0.000 |
| 3.00 | -0.120 | 0.023 |
| 6.00 | -0.240 | 0.047 |
| 9.00 | -0.350 | 0.071 |
| 12.00 | -0.490 | 0.117 |
| 15.00 | -0.640 | 0.183 |
| 18.00 | -0.860 | 0.291 |
| 20.00 | -1.070 | 0.409 |
| 20.30 | -1.360 | 0.512 |

| **Load**  **/MPa** | **Unit 1** | **Unit 2** | **Unit 3** | **Unit 4** | **Unit 5** | **Unit 6** | **Unit 7** | **Unit 8** | **Unit 9** | **Unit 10** | **Unit 11** | **Unit 12** |
| --- | --- | --- | --- | --- | --- | --- | --- | --- | --- | --- | --- | --- |
| 4.861 | 0.001606 | 0.002679 | 0.002385 | 0.002188 | 0.002398 | 0.001829 | 0.001836 | 0.001829 | 0.002201 | 0.001664 | 0.001664 | 0.001039 |
| 4.862 | 0.001607 | 0.003048 | 0.002661 | 0.002549 | 0.002665 | 0.001947 | 0.001952 | 0.001949 | 0.0022 | 0.001667 | 0.001666 | 0.001039 |
| 4.863 | 0.001609 | 0.00351 | 0.002992 | 0.002995 | 0.002993 | 0.0021 | 0.002143 | 0.002101 | 0.002198 | 0.001669 | 0.001669 | 0.001038 |
| 4.864 | 0.001612 | 0.00407 | 0.003443 | 0.003555 | 0.003439 | 0.002283 | 0.002388 | 0.002281 | 0.002196 | 0.001671 | 0.001671 | 0.001038 |
| 4.865 | 0.001615 | 0.004748 | 0.004036 | 0.004249 | 0.004037 | 0.002512 | 0.002673 | 0.00251 | 0.002194 | 0.001673 | 0.001674 | 0.001037 |
| 4.866 | 0.001619 | 0.005592 | 0.004789 | 0.00514 | 0.004795 | 0.002798 | 0.00304 | 0.002795 | 0.002192 | 0.001676 | 0.001676 | 0.001036 |
| 4.867 | 0.001625 | 0.006691 | 0.005791 | 0.006306 | 0.005791 | 0.003167 | 0.003505 | 0.003167 | 0.00219 | 0.001679 | 0.001679 | 0.001036 |
| 4.868 | 0.001634 | 0.008162 | 0.00714 | 0.007906 | 0.007158 | 0.003665 | 0.00413 | 0.003655 | 0.002188 | 0.001681 | 0.001681 | 0.001035 |
| 4.869 | 0.001648 | 0.010075 | 0.008991 | 0.010082 | 0.008983 | 0.004311 | 0.004955 | 0.004315 | 0.002189 | 0.001683 | 0.001683 | 0.001034 |
| 4.870 | 0.001666 | 0.012719 | 0.011556 | 0.013078 | 0.011547 | 0.005191 | 0.006053 | 0.005186 | 0.002191 | 0.001686 | 0.001686 | 0.001033 |
